# Clinical efficacy of dexamethasone combined with isoniazid in the treatment of tuberculous meningitis and its effect on peripheral blood T cell subsets

**DOI:** 10.1515/med-2024-0948

**Published:** 2024-06-21

**Authors:** TianYong Hou, JianWei Wang, Liang Shi, ShuHui Fan, JingYing Li, QuanHong Wang

**Affiliations:** Department of Infection Division, Taiyuan Fourth People’s Hospital, Taiyuan City, Shanxi Province, 030053, China; Department of Comprehensive Inspection, Taiyuan Fourth People’s Hospital, Taiyuan City, Shanxi Province, 030053, China; Department of Medical Section, Taiyuan Fourth People’s Hospital, Taiyuan City, Shanxi Province, 030053, China; Department of Inspection Division, Taiyuan Fourth People’s Hospital, Taiyuan City, Shanxi Province, 030053, China; Department of Serous Cavity Tuberculosis, Taiyuan Fourth People’s Hospital, Wanbailin District, Taiyuan City, Shanxi Province, 030053, China

**Keywords:** tuberculous meningitis, dexamethasone, isoniazid, clinical effect, T cell subsets

## Abstract

**Objective:**

To investigate the clinical efficacy of dexamethasone (Dex) combined with isoniazid in tuberculous meningitis (TBM) and its effect on peripheral blood T cell subsets.

**Methods:**

A total of 235 patients with TBM were divided into the control group (117 cases) and the observation group (118 cases). Both groups were given conventional treatment, the control group was further given isoniazid, and the observation group was further given Dex combined with isoniazid. The therapeutic effect and improvement of clinical symptoms were evaluated, peripheral blood T lymphocyte subsets and neurological function were observed, and patients’ prognosis was evaluated.

**Results:**

The total effective rate of the observation group was higher. The recovery time of cerebrospinal fluid (CSF) pressure, CSF protein content, CSF cell count, and hospital stays in the observation group were shorter. The duration of cervicogenic headache, fever, vomiting, and coma in the observation group was shorter. CD3^+^ and CD4^+^/CD8^+^ proportions in the observation group were higher, and CD8^+^ proportion was lower. The NIHSS score and MRS score of the observation group were lower, as well as the incidence of adverse reactions.

**Conclusion:**

Dex combined with isoniazid alleviates clinical symptoms and neurological abnormalities and regulates peripheral blood T cell subsets in TBM.

## 1 Introduction

Tuberculous meningitis (TBM) is a tuberculous inflammatory change in the meninges caused by *Mycobacterium tuberculosis* invading the subarachnoid space. Besides the meninges, the lesions often involve the brain parenchyma, arachnoid, and cerebral vessels. Without timely and effective treatment in the early stage, the prognosis is poor, with mild disability or death. Drugs that can pass the blood–brain barrier and have a high concentration in cerebrospinal fluid (CSF) should be selected in the treatment plan [1]. TBM is commonly treated with intravaginal injections of isoniazid, which can not only improve isoniazid level in CSF to form a locally high-concentration bactericidal environment to kill *M. tuberculosis*, but also avoid systemic drug conversion into acetylene isoniazid through the liver, and thereby reduce liver damage and systemic adverse reactions [2]. Monotherapy, however, is ineffective and may cause adverse effects, according to relevant studies. Due to the serious sequelae caused by comorbidities, combined drug therapy is required [3,4]. Glucocorticoids have powerful anti-infection effects, inhibit inflammatory response, significantly improve brain edema, reduce mortality, and alleviate sequelae of the nervous system in TBM patients. Clinical data also show that glucocorticoids, as the main treatment plan in the symptomatic treatment of TBM, act by binding receptors to improve the transcription process of inflammatory genes, reduce inflammatory exudation and inflammatory protein synthesis, reduce the level of inflammatory factors, and promote the return of intracranial pressure and brain tissue blood supply to normal [5,6]. Based on this, the purpose of this study was to explore the clinical efficacy of dexamethasone (Dex) combined with isoniazid in the treatment of TBM and the influence on peripheral blood T cell subsets and to provide a reference for the clinical treatment of this disease.

## 2 Data and methods

### 2.1 Clinical data

A total of 235 patients with TBM from January 2018 to January 2022 were studied and were divided into the control group (117 cases) and the observation group (118 cases) by random number table method. Clinical data from both groups were not significantly different (*P* > 0.05, Table 1), as shown in Table 1.

**Table 1:** Comparison of clinical data between the two groups

Items		Observation group (*n* = 118)	Control group (*n* = 117)	*χ* ^2^	*P*
Gender	Male	71	68	0.102	0.749
	Female	47	49		
Age (years)		37.92 ± 10.51	38.78 ± 11.47	0.59	0.55
Course of disease (days)		13.54 ± 2.69	13.47 ± 2.51	0.206	0.837
Clinical manifestations	Headache	100	105	1.318	0.251
	Fever	81	85	0.454	0.5
	Nausea and vomiting	69	66	0.102	0.749
	Disturbance of consciousness	31	28	0.171	0.679
	Optic nerve injury	27	23	0.364	0.546
	Vestibular nerve injury	15	14	0.03	0.862

### 2.2 Inclusion criteria

Inclusion criteria included: (1) patients who met the diagnostic criteria for TBM [7] and were confirmed by imaging and laboratory examination; (2) patients with clinical symptoms of tuberculosis poisoning including appetite loss, fever, night sweats, fatigue, listlessness, etc.; and (3) patients with cerebral pressure higher than 1.96 kPa.

### 2.3 Exclusion criteria

Exclusion criteria included: (1) patients with serious primary diseases of the liver, heart, and kidney; (2) patients with cognitive dysfunction or accompanied by mental illness; (3) patients with malignant brain tumors; (4) patients with severe cardiac, liver, and renal dysfunction; (5) patients who are sensitive to the tested drugs; (6) patients with severe diabetes; and (7) patients with other craniocerebral diseases.

### 2.4 Methods

Patients were given conventional treatments including anti-tuberculosis chemotherapy and other measures to reduce intracranial pressure, brain edema, and infection. The control group was given isoniazid injections in the treatment enhancement period (Tianjin Jinyao Amino Acid Co., Ltd, H12020970, specification: 2 mL:100 mg). Before injection, 0.9% sodium chloride injection was diluted to <0.2%. Injection diluent of 5 or 10 mL was pumped into a sterile syringe, and the lumbar spine was punctured with a puncture needle. CSF flowed into the syringe, and all drugs were injected slowly within 3–5 min. After the injection was completed, the puncture needle was pulled out. Injections were performed 2–3 times every week.

The observation group was supplemented with intranasal injection with Dex sodium phosphate injection (Ma’anshan Fengyuan Pharmaceutical Co., Ltd, H20051748, specification: 5 mg), 5 mg/time, 2–3 times/week. Both groups were treated for 6 months.

### 2.5 Outcome measures

(1) Clinical efficacy: All patients were followed up for at least 3 months, and the consciousness recovery time and efficacy were observed. The efficacy was evaluated according to the Glasgow Coma Scale (GCS) [8]. Recovery: After treatment, the relevant signs and symptoms completely disappeared, no obvious sequelae appeared, 14 points < GCS score < 15 points. Effective: Relevant signs and symptoms were significantly improved, no sequelae appeared, GCS score < 14 points. Ineffective: Signs and symptoms were not significantly improved, and sequelae such as limb paralysis and intellectual disability appeared.
(2) Treatment effect: The recovery time of CSF pressure, CSF protein content, CSF cell count, and hospital stays were compared.
(3) Improvement of clinical symptoms: The duration of cervicogenic headache, fever, vomiting, and coma were compared.
(4) T lymphocyte subsets: Flow cytometer (EPICS-XL type, COULTER, USA) and fluorescently labeled mouse anti-human CDs (UCHT1, 13B 8.2, and B9.11 for detecting CD3^+^, CD4^+^, CD8^+^, and CD16^+^ 56^+^ mouse monoclonal antibodies, respectively). CD3^+^, CD4^+^, and CD8^+^ in peripheral blood mononuclear cells were detected, and the proportion of CD4^+^/CD8^+^ was calculated.
(5) Neurological function: Neurological function was evaluated by National Institutes of Health Stroke Scale (NIHSS) before and 3 months after treatment [9].
(6) Prognosis: Patients’ prognosis was assessed according to the modified Rankin Scale (MRS) [10]. 0 points: no symptoms at all; 1 point: symptoms appeared without disability; 2 points: mildly disabled, able to take care of oneself; 3 points: moderately disabled, in need of help; 4 points: severely disabled, unable to take care of oneself; 5 points: severely disabled, bedridden, in need of care; 6 points: death.

### 2.6 Data analysis

Data were processed by SPSS 22.0 software. Enumeration data were expressed as percentage and tested by *χ* ^2^ test. Measurement data were expressed as (±s) after the normality test and tested by *t*-test. *P* < 0.05 meant that the difference was statistically significant.


**Ethical approval:** The present study was approved by the Ethics Committee of Taiyuan Fourth People’s Hospital. All procedures were performed in accordance with the ethical standards of the Institutional Review Board and The Declaration of Helsinki, and its later amendments or comparable ethical standards.
**Informed consent:** Written informed consent was provided by all patients prior to the study start.

## 3 Results

### 3.1 Clinical efficacy

The total effective rate of the observation group was higher than that of the control group (*P* < 0.05, Table 2).

**Table 2:** Comparison of clinical efficacy between the two groups (cases, %)

Groups	*n*	Recovery	Effective	Ineffective	Total effective rate
Observation group	118	37	71	10	91.53
Control group	117	30	65	22	81.2
*χ* ^2^					5.328
*P*					0.023

### 3.2 Treatment effects

**Figure 1:**
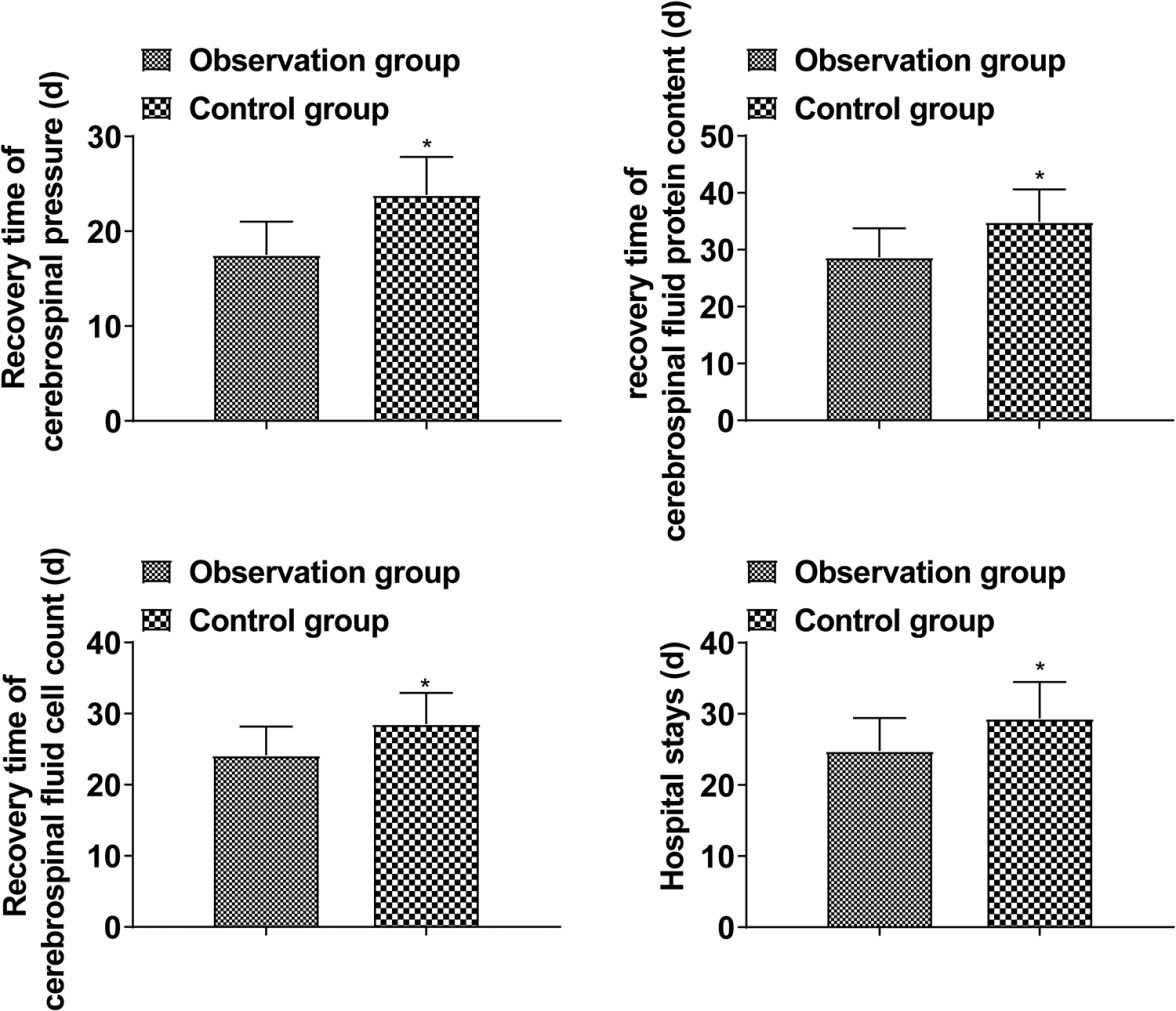
Treatment effect.

The recovery time of CSF pressure, CSF protein content, CSF cell count, and hospital stay in the observation group was shorter than those in the control group (*P* < 0.05, Figure 1).

### 3.3 Improvement of clinical symptoms

**Figure 2:**
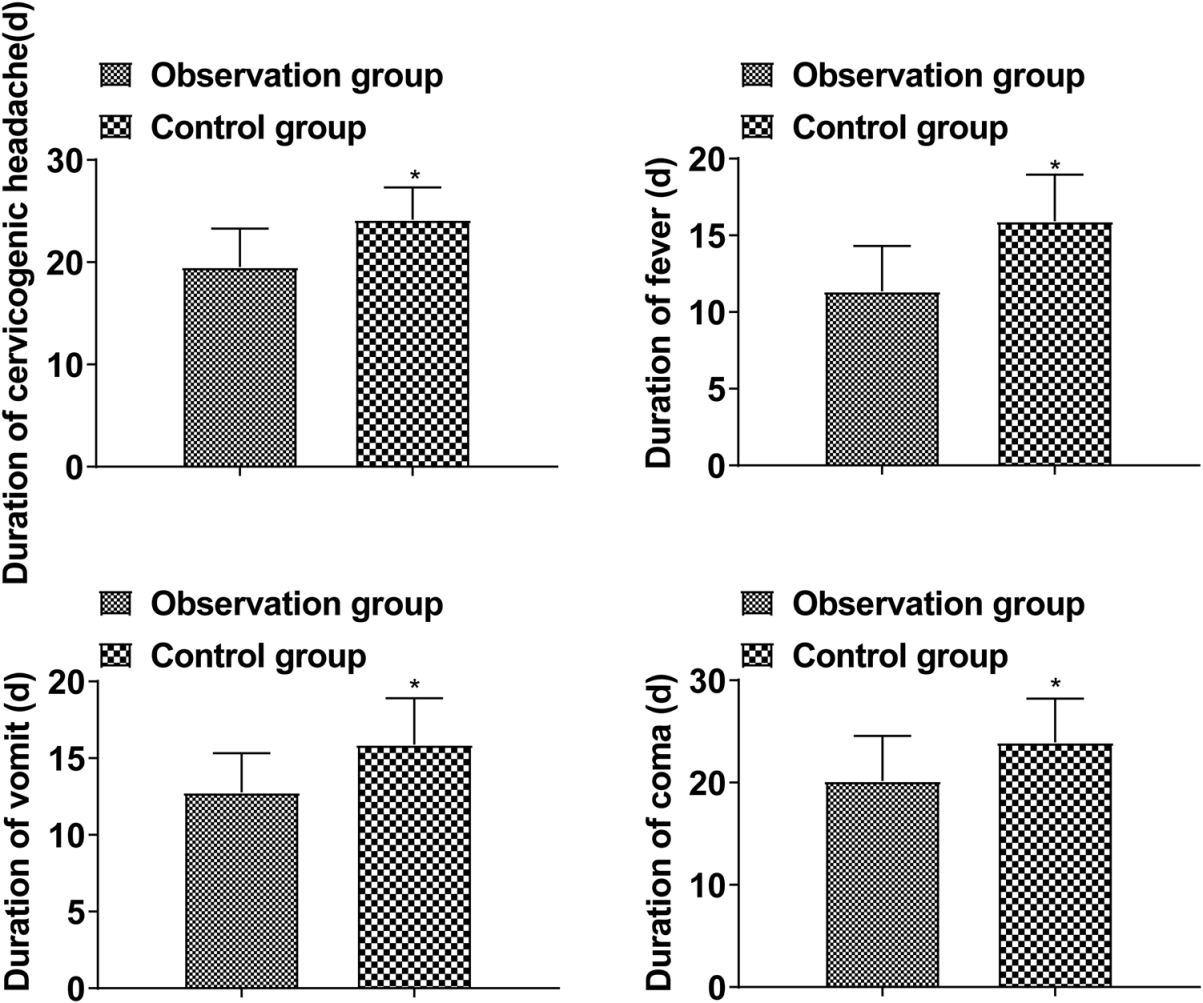
Improvement of clinical symptom.

The duration of cervicogenic headache, fever, vomiting, and coma in the observation group was shorter than that in the control group (*P* < 0.05, Figure 2).

### 3.4 Neurological function

**Figure 3:**
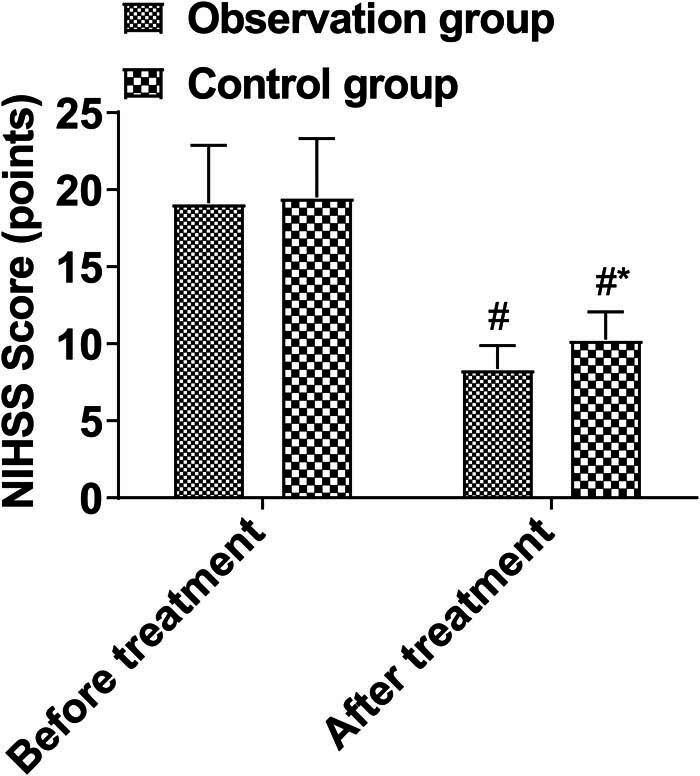
Neurological function.

Pre and post-treatment NIHSS scores did not differ significantly between the groups (*P* > 0.05). There was a reduction in NIHSS scores in both treatment groups after treatment, and the observation group scored lower than the control group after treatment (*P* < 0.05, Figure 3).

### 3.5 T cell subsets in peripheral blood

**Figure 4:**
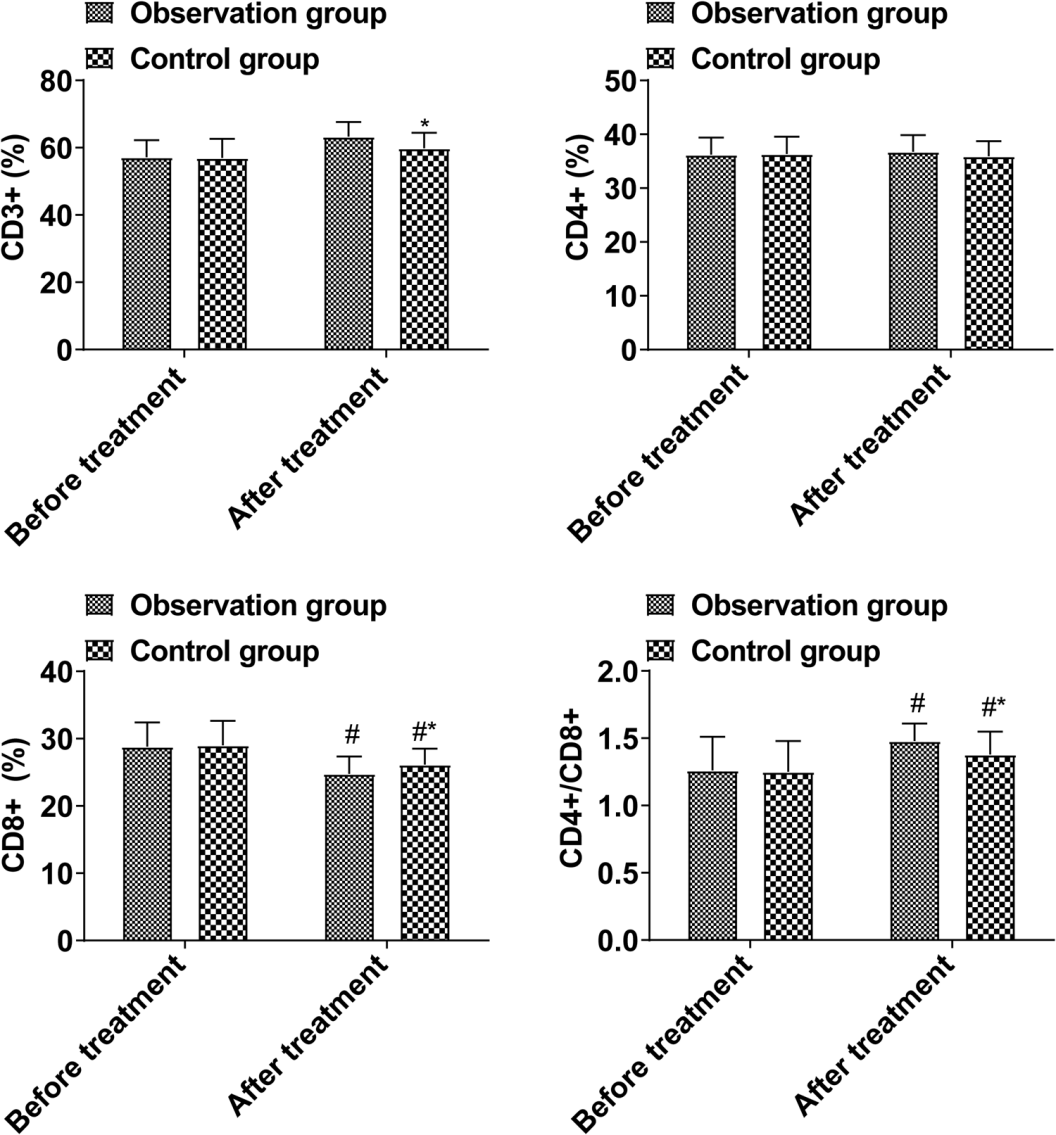
Peripheral blood T cell subsets.

A comparison of the proportions of CD3^+^, CD4^+^, CD8^+^, and CD4^+^/CD8^+^ T cells in peripheral blood before and after treatment revealed no significant difference (*P* > 0.05). After treatment, the proportion of CD3^+^ and CD4^+^/CD8^+^ in the observation group was higher than that in the control group, and that of CD8^+^ was lower (*P* < 0.05, Figure 4).

### 3.6 Prognosis

**Figure 5:**
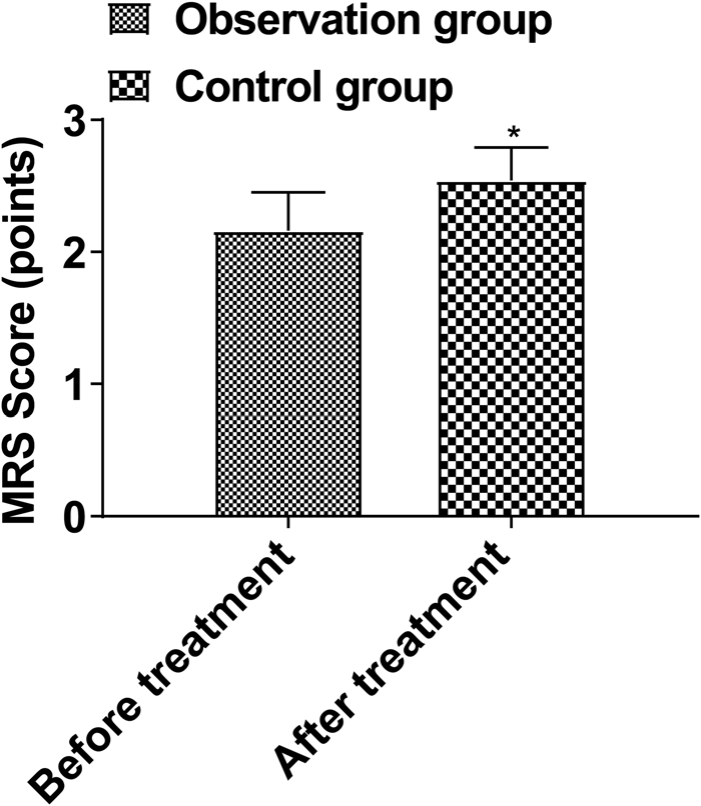
Comparison of prognosis. Note: Compared with before treatment, #*P* < 0.05; compared with the control group, *P < 0.05.

MRS scores in the observation group (2.16 ± 0.29) were lower than those in the control group (2.54 ± 0.25) (*P* < 0.05, Figure 5).

### 3.7 Adverse reactions

Isoniazid is metabolized by the liver to acetyl isoniazid in the body, which may cause liver dysfunction or hepatitis. In addition, the experiments of Girgis et al. [11] showed that the use of isoniazid may cause mild adverse reactions and neurological problems, such as neuropathy, usually characterized by low intracranial pressure headache and numbness in the hands and feet. In addition, as mentioned in previous studies, Dex, as a corticosteroid, may cause gastrointestinal problems, such as gastric ulcers and gastrointestinal bleeding, bone problems, osteoporosis, joint pain, and other conditions [12,13]. The observation group showed a lower incidence of adverse reactions than the control group (*P* < 0.05, Table 3).

**Table 3:** Comparison of the incidence of adverse reactions between the two groups (cases, %)

Groups	*n*	Abnormal liver function	Low intracranial pressure headache	Water–electrolyte balance	Lumbago and backache	Total incidence
Observation group	118	0	1	1	0	1.69
Control group	117	2	3	3	1	7.69
*χ* ^2^						4.736
*P*						0.03

## 4 Discussion

TBM is a non-suppurative inflammation of the meninges caused by *M. tuberculosis*. In addition to the cerebral pial, the arachnoid, cerebral parenchyma, and cerebral vessels are also often involved. *M. tuberculosis* does not produce toxins or enzymes, and its mechanism of causing tissue damage is mainly associated with toxic reactions and delayed hypersensitivity reactions caused by cellular immune responses [14]. Therefore, timely and appropriate treatment can effectively avoid the recurrence of inflammation.

Isoniazid is a commonly used bactericidal drug, which can inhibit the acid synthesis of *M. tuberculosis* and effectively improve cell wall rupture. Clinical data show that intramuscular injection of drugs has the advantage of targeted delivery, increasing local drug concentration, avoiding first-pass effects, and reducing hepatic metabolism of drugs. Intrathecal injection of isoniazid into the lesion can directly act on the cerebral pial and intima, effectively increasing the concentration of drugs in the local cerebral vessels and the nerves in subarachnoid space [15,16]. Although isoniazid has a strong ability to penetrate the blood–brain barrier, the clinical benefits of high doses of isoniazid are still unclear. Glucocorticoid adjuvant treatment of TBM is recognized, which can not only reduce the symptoms of severe poisoning, but also reduce brain edema, reduce exudation to reduce cerebral arteritis, prevent encephalomalacia, and prevent fibrous tissue hyperplasia [17]. This study found that the total effective rate in the observation group was higher than that in the control group, and the recovery time of CSF pressure, CSF protein content, CSF cell count, and hospital stay were shorter than that in the control group, indicating that Dex combined with isoniazid therapy for TBM patients could improve the therapeutic effect. This is mainly because intrathecal therapy combined with isoniazid and Dex can rapidly increase the concentration of isoniazid in CSF, avoid the reduced efficacy of isoniazid after systemic administration through liver metabolism, and form a local high concentration of killing tuberculosis bacteria environment in which inflammation is inhibited more effectively, the generation of CSF is reduced, inflammatory exudation and meningeal adhesion/thickening are suppressed, CSF circulation and absorption are improved, thereby effectively reducing hydrocephalus and intracranial pressure.

At present, it is believed that the neurological function injury caused by TBM may be caused by tuberculous bacteria in the nervous system, which can cause exudative and proliferative lesions. Exudates deposited in the subarachnoid space and lateral fissure cistern can spread and wrap around the brain stem, thereby oppressing the cerebral nerves, especially optic chiasma and peduncle, so optic nerve and optic chiasm are surrounded by the exudate of the basal cistern. The granulation tissue of the basal cistern proliferates and infiltrates the optic nerve, causing neurilemmitis. Some cases affect the AB circulation, resulting in ventricle foramen and aqueduct stenosis, resulting in obstructive hydrocephalus. Excessive AB secretion results in communicating hydrocephalus, cerebral nerve stretching, and paralysis. Sometimes occlusive arteritis occurs when brain stem vessels are involved, and encephalomalacia leads to brain nerve damage. Late intracranial hypertension and cerebral hernia can also damage cerebral nerves [18,19]. The results of this study showed that the duration of cervicogenic headache, fever, vomiting, and coma in the observation group was shorter than in the control group, and the NIHSS score after treatment was lower, indicating that Dex combined with isoniazid in the treatment of TBM can alleviate clinical symptoms and improve neurological function. This is mainly because the combined treatment of isoniazid has an inhibitory effect on the activity and reproduction of *M. tuberculosis*, which can effectively improve the bactericidal effect. Dex, as a preventive drug for inflammation and allergic reactions, can promote the reduction of membrane permeability and inflammatory response, so the combined treatment can effectively protect the cranial nerve and spinal nerve [20,21].

Clinical data show that in the course of treatment, toxic reactions and delayed hypersensitivity reactions caused by cellular immune response induced by *M. tuberculosis* are more likely to lead to death of patients than inflammation itself [22,23]. In this study, it was found that the proportions of CD3^+^ and CD4^+^/CD8^+^ in the observation group were higher than those in the control group after treatment, and the CD8^+^ was lower, indicating that Dex combined with isoniazid could regulate the peripheral blood T cell subsets of patients with TBM and improve the immune function. The reason is that intrathecal injection of Dex can effectively improve the blood–brain barrier permeability, reduce inflammatory exudation and improve vascular permeability at the lesion site, improve immune response, reduce intracranial hypertension, and prevent adverse reactions. Intrathecal injection of isoniazid can increase the concentration of drugs in CSF to improve bactericidal effect [24–26]. The combination of the two drugs can further reduce inflammatory response and improve immune function. In addition, this study found that the MRS score and the incidence of adverse reactions in the observation group were lower than those in the control group, indicating that combined treatment would not increase adverse reactions and could improve the prognosis of patients, which was mainly related to the bactericidal and anti-inflammatory effects of combined treatment. Isoniazid is highly selective to *M. tuberculosis* and has a strong antibacterial effect. A higher concentration of isoniazid has a strong bactericidal effect on bacteria in the breeding period, and can directly kill *M. tuberculosis* in the chest cavity or pleural effusion. Injection of isoniazid into the chest cavity can increase the local drug concentration, strengthen the sterilization effect, and thus reduce the exudation of the pleural cavity. Glucocorticoids can increase capillary permeability, avoid the aggregation of inflammatory factors, inhibit poison metabolism, protect mitochondrial cells, effectively reduce the number of CSF white blood cells, and combine antigens and antibodies. Finally, it can effectively avoid hydrocephalus in patients and reduce brain edema [23,27,28].

Suárez et al. found that after Dex was tapered or discontinued, patients developed generalized seizures or CNS damages [29]. Due to time and resource constraints, the postoperative follow-up of patients in this study was too short to adequately assess the efficacy of Dex in combination with isoniazid in the long-term treatment of TBM, and we plan to extend the follow-up period in future studies to have assessed the efficacy and safety of Dex in combination with isoniazid in clinical use. Although 235 patients were included in this study, the sample size was relatively small, and it cannot be ruled out that a larger trial may differ from our hypothesis. We will conduct a larger randomized controlled trial in future studies and develop a personalized drug regimen based on the actual situation of the patients, in order to improve the statistical efficacy and generalizability of the results of the study, and to achieve the best therapeutic efficacy and the smallest side effects. We will continue to explore the long-term efficacy of the combination of Dex and isoniazid in the treatment of TBM in the future, and continue to monitor the maximum benefit of the combination therapy and intervene in adverse effects.

In summary, Dex combined with isoniazid has high clinical efficacy in the treatment of TBM, which can reduce clinical symptoms and neurological abnormalities and regulate peripheral blood T cell subsets to improve immune function and patients’ prognosis.
